# Temporary transarticular stabilization with locking compression plate for the treatment of traumatic glenohumeral luxation in 10 dogs

**DOI:** 10.1111/vsu.70106

**Published:** 2026-04-15

**Authors:** Alexandros Bourbos, Francesco Piana, Alex Belch, Luca Vezzoni

**Affiliations:** ^1^ Langford Veterinary Hospital University of Bristol Bristol UK; ^2^ Veterinary Clinic Vezzoni Cremona Italy

## Abstract

**Objective:**

The aim of this study was to describe the surgical technique, and the short to medium clinical outcome of dogs treated with temporary glenohumeral joint (GHJ) stabilization following traumatic luxation.

**Study Design:**

Short case series.

**Methods:**

Medical records of dogs presented with GHJ luxation and treated with a temporary transarticular locking compression plate and screws were reviewed. Signalment, clinical signs, direction and duration of the luxation, diagnostic imaging, operative technique, complications and clinical outcome were reviewed. Liverpool Osteoarthritis in Dogs (LOAD) and Canine Orthopedic Index (COI) questionnaires were completed by the owners at 6 months after implant removal.

**Results:**

Clinical and radiographic findings were documented for 10 client‐owned dogs at a mean follow‐up of 42.5 days when implants were removed through a minimally invasive procedure. Two dogs exhibited minor complications identified through radiographs performed at the follow‐up appointment consisting of screw loosening or breakage. These did not affect the clinical outcome. Temporary GHJ stabilization resulted in return stability and range of motion for all dogs. At 6 months 9/10 dogs returned to complete function while one died for other causes. In one case, luxation recurred 8 months postoperatively for which the dog underwent glenohumeral joint arthrodesis.

**Conclusion:**

Temporary transarticular GHJ stabilization is a possible treatment option in cases of traumatic glenohumeral joint luxation. Further studies are required to determine the minimum immobilization time necessary to achieve joint stability, to assess the long‐term complication rate and the possible risks associated with this technique in a controlled population.

## 1 INTRODUCTION

Luxation of the glenohumeral joint (GHJ) is an uncommon orthopedic condition that is most often reported in small‐breed dogs, although it can occur in large breeds.^1^ It has been proposed that mechanisms leading to instability and luxation include abnormality of the glenoid concavity, torn or avulsed glenohumeral ligament, hypoplasia, or dysplasia of the GHJ, and fracture of lesser tubercle resulting in lack of concavity compression between the glenoid and humeral head.^2^ While other biomechanical concepts have been reported in human literature which include, dynamic muscle imbalance and abnormal angulation of the glenoid, and disruption of the capsuloligamentous restraints^3^, ^4^ comparable data have not documented. Medial luxation has been more often described, while lateral and cranial instability are less frequently reported, and are usually associated with a traumatic etiology.^5^, ^6^, ^7^, ^8^ Numerous treatments have been described to stabilize GHJ instability or luxation. These can be divided into conservative and surgical methods. The former includes closed reduction and external coaptation with a Velpeau or Spica sling and physical rehabilitation. More severe injuries may be better treated by open reduction and surgical stabilization. Various surgical stabilization techniques have been proposed to reconstruct or augment the joint capsule and glenohumeral ligaments; these include using radiofrequency‐induced thermal capsulorrhaphy,^9^ transposition of the biceps brachii^10^, ^11^ or supraspinatus tendon,^12^, ^13^ imbrication of the subscapularis muscle tendon in cases of glenohumeral instability,^14^ suture stabilization^12^, ^13^, ^14^, ^15^ and prosthetic ligament repair.^16^ Approaches which involve tendon transposition are proposed to alter the biomechanics of the GHJ causing incongruency of articular surface and subsequent degenerative joint disease.^2^, ^17^ Bicipital tendon transposition and a modified Campbell glenohumeral encircling prosthesis has been described in a thoracic limb amputee dog.^18^ Transarticular pinning as a stabilization method has been proposed in cases of marked instability^19^ and GHJ arthrodesis as a salvage procedure in dogs diagnosed with significant chondromalacia, glenoid dysplasia, non‐reconstructable fractures, recurrent luxation or end‐stage osteoarthritis.^2^, ^20^, ^21^ An alternative technique for GHJ stabilization, which aims to maintain the soft tissues and preserve the articular surfaces and ultimately the joint function through a temporary joint immobilization, has shown satisfactory functional results in one case report.^22^ While this case report demonstrated a promising functional result using temporary joint stabilization with a locking compression plate (LCP; DePuy Synthes, West Chester, Pennsylvania) for GHJ luxation, further case series evaluating this technique in a larger group of dogs are lacking. The aim of the present study was to report the use of an LCP plate in a larger cohort of dogs, specifically examining the complications associated with the procedure and the long‐term functional outcomes.

## 2 MATERIALS AND METHODS

Medical records from two referral veterinary hospitals were searched and dogs that were presented for treatment of GHJ luxation were retrospectively reviewed. Data retrieved included signalment, type and direction of luxation, previous treatment, concurrent orthopedic diseases, implants that were used for the temporary GHJ stabilization, concomitant surgical procedures, subjective lameness grade at presentation and post implant removal. Owners' perception of outcomes was assessed through the Liverpool Osteoarthritis in Dogs (LOAD) and Canine Orthopedic Index (COI) questionnaires^23^ at 6 months after implant removal. Complications, rate and time frame of their resolution were classified as previously described.^24^

### 2.1 Preoperative evaluation

Mediolateral and craniocaudal radiographs were obtained for all dogs as part of the initial assessment. Computed tomography (CT) was considered based on the suspicion that an articular fracture could be present due to the nature of the trauma, road traffic accident, and the severe pain on the orthopedic examination of the affected GHJ and CT was preferred over radiographic examination to provide more details.

### 2.2 Surgical technique

All dogs were premedicated with methadone (Comfortan; Dechra Veterinary Products) 0.3 mg/kg IV and general anesthesia was induced with propofol (PropoFlo, Zoetis Ltd, UK) 2–4 mg/kg IV. Anesthesia was maintained with 2% isoflurane (Isoflurane Vet, Boehringer Ingelheim Animal Health Ltd, UK) in oxygen. Cefuroxime (Zinacef, GlaxoSmithKline, UK) 20 mg/kg IV was given 30 min prior to skin incision and continued every 90 min during the surgery. All dogs were placed in lateral recumbency, with the affected limb uppermost, and, if needed, an open reduction was achieved through a cranio‐lateral approach as previously described.^25^ The first two dogs of the study were treated with an open surgical approach; additional retraction of the brachiocephalicus and the pectoralis muscles were performed to assess the medial aspect of the GHJ. In both cases the medial gleno‐humeral ligament (MGHL) was found to be completely ruptured, and this was repaired with polydioxanone suture in a simple interrupted suture pattern. Injury to the static and dynamic stabilizers can cause joint laxity or subluxations or in severe cases luxation of the GHJ, The aim of ligament repair was to provide additional stability to our temporary repair. Due to complete rupture of the MGHL there was not sufficient tissue to perform a three‐loop pulley or other any reconstruction suture pattern. We aimed to oppose the edges of the MGHL and expect fibrous tissue to form while maintaining the GHJ temporary immobilized with the transarticular plate. The simple interrupted pattern was selected to approximate the ligamentous tissue in these two cases, and the goal was to restore anatomical alignment rather than recreate the original fiber orientation.

**FIGURE 1:**
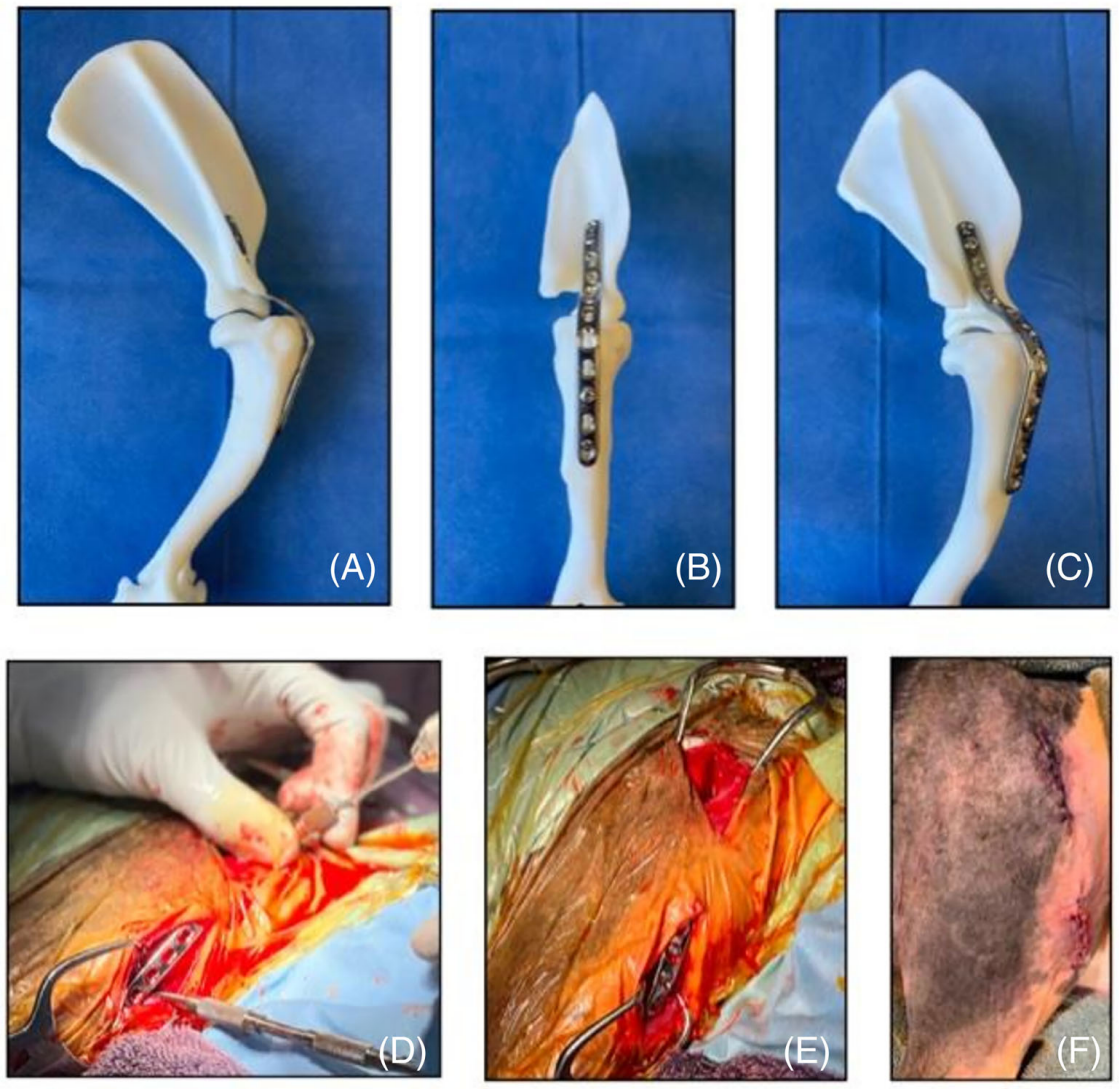
(A–C) latero‐medial, cranio‐caudal and oblique views of scapula and humerus plastic bone models with applied LCP plate; (D–F) intraoperative views of MIPO application of a 2.7 mm LCP in case 8.

**FIGURE 2:**
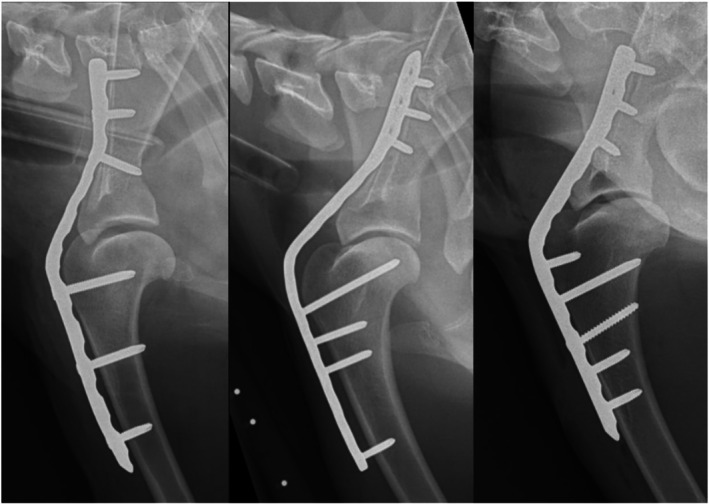
From left to right postoperative medio‐lateral radiographs of the glenohumeral joint of cases 1, 2 and 3.

The remaining dogs underwent a minimal invasive plate osteosynthesis (MIPO) (Figure 1). For the MIPO cases a small incision was performed at the cranial aspect of the scapular spine and the supraspinatus muscle was partially elevated from the cranial aspect of the scapular spine with a Freer periosteal elevator. The suprascapular nerve was identified and protected. A similar size incision was performed at the proximal aspect of the humerus and the bone was exposed after caudal retraction of the deltoideus muscle. The precontoured bone plate was inserted from the proximal humerus exiting on the cranial aspect of the scapular spine. An LCP plate was preoperatively contoured for all dogs, based on the standing angle of the contralateral GHJ which ranged between 115° and 150°. Intraoperatively, the plates were readjusted to allow for adequate positioning on the bone. The plate was then secured with a combination of locking and non‐locking screws, with a minimum of six cortices per bone fragment (Figures 1 and 2). With the non‐locking screw, which was placed first, we aimed to bring the plate as close as possible in contact with the bone to reduce the strain on the implant. The surgical wound was closed routinely in layers after copious lavage. Postoperative radiographs were obtained to assess the joint reduction and implant positioning (Figure 2). In all dogs, postoperative analgesia was provided with methadone 0.2 mg/kg and meloxicam (Metacam, Boehringer Ingelheim Animal Health Ltd) 0.1 mg/kg for the next 48 h. Two days after surgery the dogs were discharged, and analgesia was provided with oral paracetamol/codeine (Pardave‐V, Dechra Veterinary Products) 10 mg/kg three times daily for 5 days and meloxicam 0.1 mg/kg once daily for 14 days; cephalexin (Cephacare, Animal Care Ltd, UK) 20 mg/kg was prescribed twice daily for 5 days based on the surgeon's preference. Strict crate rest was used for the first week after the temporary plate application. After this period short lead walking was recommended. At the surgeon's discretion, transarticular plate removal was planned between 4 and 8 weeks postoperatively. Limiting the immobilization to a shorter time frame (6–8 weeks) seems to have negligible deleterious effects on articular cartilage. Based on this, we arbitrarily planned the removal of the transarticular plate at this time frame.

**FIGURE 3:**
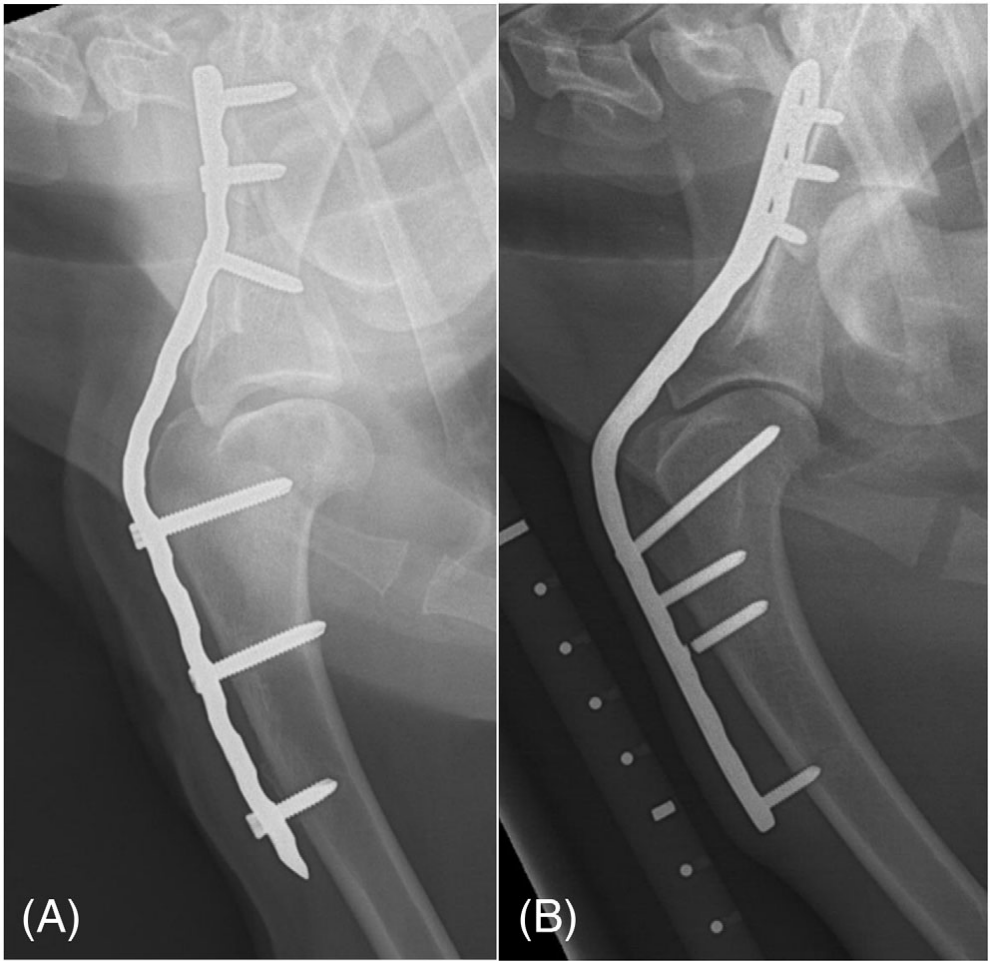
(A) follow‐up medio‐lateral radiograph of case 1 showing mild backout of the three distal screws; (B) follow‐up medio‐lateral radiograph of case 2 showing fracture of the penultimate distal screw and failure at the bone‐screw interface of the most distal screw, resulting in backout of the distal portion of the plate.

All cases underwent general anesthesia to facilitate implant removal through a limited cranio‐lateral approach over the screws proximally on the scapular spine and distally on the proximal aspect of the humerus (Figure 3). Immediately after plate removal physical activity was restricted to inhouse confinement and short leash walks. Two weeks after implant removal clients were instructed to progressively increase the activity and to start an in‐home exercise program under the guidance of a rehabilitation practitioner. Exercises included passive range of motion as well as specific weightbearing exercises.

### 2.3 Clinical evaluation

Clinical evaluation was performed using orthopedic and radiographic examinations at the following time points: preoperatively, immediately postoperatively, at the time of implant removal. All clinical examinations were performed by the attending surgeon and included orthopedic examination and gait assessment. Subjective evaluation of lameness was used to determine clinical outcome using the following five‐point scoring system: 1 = no lameness; 2 = mild intermittent weightbearing lameness; 3 = persistent moderate weightbearing lameness; 4 = persistent severe weightbearing lameness, with or without non‐weightbearing lameness; 5 = persistent non‐weightbearing lameness. At 6 months post implant removal, owners were contacted to complete LOAD and COI questionnaires.

## 3 RESULTS

**FIGURE 4:**
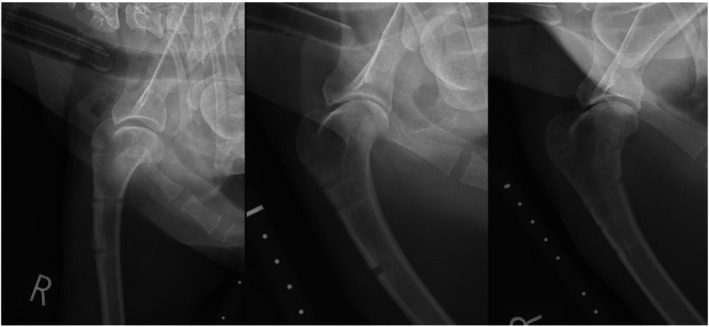
From left to right postoperative medio‐lateral radiographs of the scapulo‐humeral joint after implant removal of cases 1, 2 and 3.

Temporary transarticular GHJ stabilization was performed in 10 dogs (10 GHJ). All dogs met the inclusion criteria. Breeds were Whippet (7 dogs), and one each of Norwegian Elkhound, Dobermann Pinscher and Greyhound cross (Table 1). There were five spayed females, four neutered males and one intact male. The mean age at the surgery was 6.4 years (SD: 2.8; range 2.1–9.10 years). The mean bodyweight of the dogs was 13.7 kg (SD: 4.6; range: 5–21.8 kg). GHJ luxation was lateral in nine dogs and medial on one dog. Overall, six 3.5 mm LCP, three 2.7 mm LCP and one 2.0 mm LCP were used; sizes were decided based on the bone diameter and weight of the dogs (Table 1). The most distal screw on the humerus was intentionally placed monocortical in 9/10 cases to prevent stress rising and avoid humeral fracture. Conservative management consisting with a combination of rest, bandaging and non‐steroidal anti‐inflammatory drugs was previously initiated at the referring veterinary clinics for all dogs and ranged between 3 and 81 days (mean 25 days). All dogs had been non‐responsive to conservative management with recurrence of the GHJ luxation and lameness. The cause of the injury was a road traffic accident in two cases and a non‐specific trauma while running or playing off leash in the remaining dogs. One dog (case 2) presented with concurrent orthopedic injuries consisting of a comminuted open fracture, Gustillo‐Anderson type III B of the right tibia and fibula. The orthopedic examination revealed a non‐weightbearing lameness on the affected thoracic limb and painful reactions on extension and flexion of the GHJ in all cases. Postoperative radiographic assessment confirmed satisfactory reduction and alignment of the GHJ and implant positioning in all cases (Figure 3). The postoperative GHJ angle varied between 114° and 156°. A spica splint was applied to minimize swelling and provide additional support of the operated limb in one dog which was removed 3 days postoperatively. The rest of the dogs had no bandage placed to the operated forelimbs. All dogs were weightbearing on the operated limb and walk on the lead the day after surgery. In all cases, after GHJ plate application, postoperative management included crate rest and lead walks for 5–8 weeks. In addition, a veterinary physiotherapist instructed the owners with a plan to perform passive range of motion of the carpus and the elbow joint and weightbearing exercises. All dogs were clinically evaluated at the scheduled day of implant removal, a mean of 42.5 days (SD:7.5; range: 35–64 days). Radiographic control was performed prior to the plate removal in all cases. Implant related complications were identified in two cases and classified as minor, based on previous classification.^24^ In one case, implant failure with migration of the three screws placed in the humerus occurred; in another case, fracture of the most distal screw resulting in pullout of the distal portion of the plate was evident (Figure 4). After plate removal, the GHJ appeared to be stable during abduction, adduction and craniocaudal drawer test in all cases. Postoperative radiographs identified a congruent GHJ. The non‐affected contralateral GHJ had significantly greater range of motion in comparison with the affected one at subjective clinical examination. Hydrotherapy was initiated in 9/10 dogs following suture removal, which occurred between 10 and 14 days after implant removal. To determine long‐term outcomes, LOAD and COI questionnaires were completed via email or telephone conversation by 9/10 owners 6 months postoperatively. One dog (case 5) had died for causes not related to this orthopedic injury and its owners were not contacted. One dog (case 8), 8 months after implant removal, had a recurrence of GHJ luxation while running off‐lead and underwent a GHJ arthrodesis.

**TABLE 1:** Signalment, luxation and surgical details, complications and questionnaires.

Case	Breed	Sex	Age	Weight (kg)	Side	Luxation	Cause	Conservative management	Imaging preop	Plate (LCP)	Complications	Plate removal (days)	LOAD n/52	COI n/48
1	Norwegian Elkhound	FN	9 year 10 month	18	R	Lateral	RTA	Rest, analgesia	X‐rays	3.5 mm 11 holes	3 screws loosening	64	1	1
2	Whippet	ME	2 year 2 month	12.3	R	Lateral	RTA	Rest, analgesia	X‐rays	2.7 mm 10 holes	1 screw fracture, distal plate pullout	36	2	3
3	Greyhound X	FN	4 year 6 month	21.8	R	Lateral	Running	Rest, analgesia	CT scan	3.5 mm 11 holes	No	42	0	0
4	Whippet	MN	7 year 3 month	12.8	R	Lateral	Running	Rest, Spica splint analgesia	CT scan	2.7 mm 12 holes	No	40	1	1
5	Whippet	FN	9 year 3 month	10	R	Lateral	Playing	Rest, analgesia	X‐rays	2.7 mm 14 holes	No	42	na	na
6	Whippet	MN	4 year 8 month	18.6	R	Lateral	Playing	Rest, Spica splint, analgesia	CT scan	3.5 mm 11 holes	No	35	0	0
7	Whippet	ME	2 year 1 month	14	L	Lateral	Playing	Rest, analgesia	CT scan	3.5 mm 11 holes	No	40	0	0
8	Whippet	FN	8 year	10	R	Lateral	Running	Rest, analgesia	X‐rays	2.7 mm 14 holes	Reluxation 8 months post implant removal	42	2	2
9	German Pinscher	MN	8 year	5	R	Medial	Playing	Rest, analgesia	X‐rays	2.0 mm 12 holes	No	42	2	2
10	Whippet	FN	9 year 10 month	14.6	L	Lateral	Running	Rest, Spica splint, analgesia	CT scan	3.5 mm 12 holes	No	42	2	3

Abbreviations: CT, computed tomography; FN, female neutered; LCP, locking compression plate; ME, male entire; MN, male neutered; RTA, road traffic accident; NA, not applicable.

## 4 DISCUSSION

Traumatic luxation of the GHJ is a relatively uncommon orthopedic condition with previous studies presenting various surgical options for its treatment which included a relatively small number of dogs, and data for long‐term outcomes are lacking. Medial luxation represents the greater proportion of this injury in the literature, resembling 75% of cases.^1^ Lateral and cranial luxation are rarely reported, and more often documented in large breed dogs and usually due to traumatic causes.^26^ Interestingly, in our case series, medial luxation was reported in only one case; however, 9/10 dogs had lateral luxation following direct or indirect traumas while running. The glenoid cavity provides limited coverage to the humeral head, therefore the tendons of the periarticular muscles (supraspinatus, infraspinatus, subscapularis, biceps brachii, coracobrachialis and teres minor) act as a dynamic stabilizer of the GHJ.^27^ In a previous study, ex vivo transection of the biceps brachii resulted in increased cranial and lateral translation when the GHJ is in either a neutral or flexed position,^28^ while a biomechanical study has shown that in full extension the joint capsule and the medial and lateral glenohumeral ligaments provide stability to the GHJ.^29^ Many surgical techniques to stabilize the GHJ were developed based on these biomechanical principles and involved reconstruction or augmentation of the joint capsule and glenohumeral ligaments.^30^, ^31^ Specifically, the transposition of the biceps brachii tendon has been described for mediolateral and cranial luxation,^10^ while the imbrication of the subscapularis muscle tendon of insertion and the supraspinatus tendon transposition were reported for glenohumeral instability.^13^, ^14^ Those surgical treatments can result in some degree of alteration of GHJ biomechanics, incongruency of articular surface and subsequent degenerative joint disease.^2^ Suture augmentation has been reported to be a viable alternative, as it might offer a better biomechanical preservation of the joint. One study that clinically evaluated the use of prosthetic medial glenohumeral ligament repair in 10 dogs found that this technique provided good medial stability with a good congruency and less alteration of normal anatomy in the majority of dogs.^16^ More recently, an experimental study conducted in a canine model found that the rotator cuff can be repaired using a poly‐L‐lactide device which might offer a functional benefit and decrease the failure rate.^17^ Glenohumeral joint arthrodesis has been indicated as a reasonable solution in severe cases where significant chondromalacia or erosion is evident at the level of the humeral head.^2^, ^20^ Various complications have been described after GHJ stabilization.^15^, ^20^, ^21^ Conservative management with joint immobilization through external coaptation may allow for soft tissue healing and scar tissue formation, providing adequate joint stability, without the need of a surgical procedure. The main drawback of the conservative management is the high complication rate reported with bandages,^32^, ^33^ associated with the risk of inadequate stabilization. The technique reported in our study aims to provide the same function as external coaptation, with the plate providing buttress stabilization enabling enough fibrous tissue formation of the capsuloligamentous and the musculotendinous tissues to achieve long‐term stability. The postoperative complications encountered in our cases included screw loosening or breakage; in one case this caused a partial pullout of the distal portion of the plate. These types of complications could be secondary to the rigidity of the implant and to the increased stress occurring to the most distal screws. These are likely under high stress due to the constant movements of the distal humerus during ambulation. Also, with this temporary stabilization technique there may be inadequate load sharing occurring between implants and bones, as the glenoid and humeral head are not in close contact; this may predispose to an increased stress to the implants compared to arthrodesis constructs. Therefore, to reduce the stress on the most distal screws, it may be sensible to try and minimize the gap distance between bone and plate. Increasing the numbers of screws (cortices) may also increase the stability of the construct. Overall, these complications did not cause any significant clinical deterioration in any of the dogs as the owners did not notice any change in lameness or level of comfort. All of these were indeed identified at the time of the follow‐up appointment which coincided with the prescheduled implant removal procedure. These complications did not influence the clinical outcome as no further intervention or different treatment was deemed necessary. However, in one case luxation recurred while running 8 months after implant removal; this patient did not have any prior implant related complications; this dog subsequently underwent GHJ arthrodesis which could be considered a catastrophic complication.^24^ However, given the long interval since surgery, the dog's previous spontaneous luxation while running, and the similar nature of reluxation in the same way, it is difficult to establish a direct link between the surgical technique and this complication.

There are potential concerns with this technique as two surgeries are necessary. However, implant removal is achieved through minimally invasive technique where two small incisions are made, one over the humeral and one over the scapula screws. This avoids disruption of the periarticular fibrosis that may be contributing to the GHJ stability and has the benefit of decreasing anesthesia and surgery time. The main benefit of this technique is the fact that the GHJ is preserved and therefore full range of motion and ultimately return to function can be expected, without altered joint biomechanics. As previously reported with this technique,^22^ the clinical outcomes were satisfactory in our cases; all the joints were found clinically stable with reduced range of motion in comparison to the contralateral thoracic limb at the time of implant removal. Also, at 6 months after surgery all dogs for which LOAD and COI were completed (9/10), returned to full function, including those which had minor postoperative complications. Although generally protracted immobilization of a joint may lead to ankylosis with obliteration of the joint space as well as degenerative changes of the articular cartilage and to muscle atrophy,^34^ other studies have identified that if the animal is permitted to ambulate after a short immobilization period, rapid and complete reversal of cartilage thickness, histologic changes, proteoglycan concentration, and cartilage biomechanics can occur.^35^ The time of immobilization in this case series varied from 35 to 64 days due to surgeons discretion. No data are available on the estimated time necessary to allow for formation of scar tissue providing adequate joint stability. Further studies are necessary to identify the minimum immobilization time necessary to achieve joint stability while minimizing detrimental consequences such as cartilage degeneration. In our case series Whippets were overrepresented (7/10); breed predisposition has not been previously reported; however, due to the low number of the cases included, no definitive assumptions can be made. The study limitations are related to its retrospective nature, the number of cases included and the limited and subjective assessments of long‐term clinical outcomes. The lack of a control group of dogs treated with different implants or other methods of temporary GHJ stabilization is another limitation which does not enable us to establish any clear superiority or inferiority of LCP over other type of implants for this technique. Based on the clinical outcome at 6 months and owners‐completed questionnaires, a positive outcome and joint function can be achieved with this technique. Further investigation in a larger and more diverse population is warranted to confirm these findings and assess the long‐term success of this technique.

## 5 CONCLUSION

Temporary transarticular GHJ stabilization is a possible treatment option in cases of traumatic GHJ luxation. Further studies are required to determine the minimum immobilization time necessary to achieve joint stability, to assess the long‐term complication rate and the possible risks associated with this technique in a controlled population.

## AUTHOR CONTRIBUTIONS

All authors contributed to the design of the study. Bourbos A, DVM, DipECVS, FHEA, MRCVS and Piana F, DVM(Hons), PGDip(VCP), DipECVS, MRCVS: Performed the literature review and drafted the manuscript. Bourbos A, DVM, DipECVS, FHEA, MRCVS, Belch A, BVMS, MSc, CertAVP(GSAS), DipECVS, MRCVS and Vezzoni L, DVM, DipECVS: Were primary clinicians and operating surgeons of the cases included in the study. All authors performed data collection, analysis and provided review of the manuscript and approved the final version.

## CONFLICT OF INTEREST STATEMENT

The authors declare no conflicts of interest related to this report.
